# Lectins engineered to favor a glycan-binding conformation have enhanced antiviral activity

**DOI:** 10.1016/j.jbc.2021.100698

**Published:** 2021-04-23

**Authors:** Yasuyuki Matoba, Yuichiro Sato, Kosuke Oda, Yuta Hatori, Kinjiro Morimoto

**Affiliations:** 1 Faculty of Pharmacy, Yasuda Women’s University, Hiroshima, Japan; 2 Department of Virology, Institute of Biomedical and Health Sciences, Hiroshima University, Hiroshima, Japan

**Keywords:** X-ray crystallography, OAA-family lectins, *Pseudomonas taiwanensis* lectin, engineered lectin, antiviral activity, HA, hemagglutinin, HIV, human immunodeficiency virus, IC_50_, 50% inhibitory concentration, OAA, *Oscillatoria agardhii* agglutinin, PCR, polymerase chain reaction, PFL, *Pseudomonas fluorescens* lectin, PTL, *Pseudomonas taiwanensis* lectin, rmsd, root mean square distance, SPR, surface plasmon resonance

## Abstract

Homologues of the *Oscillatoria agardhii* agglutinin (OAA) lectins contain a sequence repeat of ∼66 amino acids, with the number of tandem repeats varying across family members. OAA homologues bind high-mannose glycans on viral surface proteins, thereby interfering with viral entry into host cells. As such, OAA homologues have potential utility as antiviral agents, but a more detailed understanding of their structure–function relationships would enable us to develop improved constructs. Here, we determined the X-ray crystal structure of free and glycan-bound forms of *Pseudomonas taiwanensis* lectin (PTL), an OAA-family lectin consisting of two tandem repeats. Like other OAA-family lectins, PTL exhibited a β-barrel-like structure with two symmetrically positioned glycan-binding sites at the opposite ends of the barrel. Upon glycan binding, the conformation of PTL undergoes a more significant change than expected from previous OAA structural analysis. Moreover, the electron density of the bound glycans suggested that the binding affinities are different at the two binding sites. Next, based on analysis of these structures, we used site-specific mutagenesis to create PTL constructs expected to increase the population with a conformation suitable for glycan binding. The engineered PTLs were examined for their antiviral activity against the influenza virus. Interestingly, some exhibited stronger activity compared with that of the parent PTL. We propose that our approach is effective for the generation of potential microbicides with enhanced antiviral activity.

Several lectins that bind to specific carbohydrate structures inhibit viral replication by interacting with their glycoproteins (1, 2). Glycans of viral surface glycoproteins play a pivotal role in many pathogenic processes, including binding and invasion into susceptible cells and immune evasion by shielding epitopes from antibody binding (3, 4, 5, 6). The structure of *N*-glycans on viral glycoproteins depends on factors such as the viral species, the host cell of origin, and position on the protein (1, 7, 8, 9, 10). Such diverse *N*-glycans on the viral surface are responsible for numerous specific biological interactions (11).

Human C-type lectins play a prominent role in the recognition of foreign pathogens, and their binding to viral glycoprotein leads to pathogen sensing and the initiation of immune responses. Some classes of them are well-known high-mannose binding lectins (*e.g.*, in the complement system, surfactant proteins A and D) (1, 12, 13). Since high-mannose glycans are predominant on viral surface proteins such as human immunodeficiency virus-1 (HIV-1) gp120 and influenza hemagglutinin (HA) protein, the antiviral activities of high-mannose binding lectins are well known (2, 14, 15, 16, 17). We previously reported that *Oscillatoria agardhii* agglutinin (OAA) family of lectins, although exogenous bacterial lectins unrelated to human C-type lectins, showed broad-spectrum antiviral activity against multiple strains of HIV and influenza virus with 50% inhibitory concentration (IC_50_) in the nanomolar range (17, 18, 19). The antiviral activity was inhibited by the addition of yeast mannan (17), indicating that binding of the lectin to high-mannose glycans is committed to the activity. Antiviral activities of high-mannose binding lectins against hepatitis C virus (20, 21), coronavirus (22, 23, 24), Ebola virus (25, 26), Japanese encephalitis virus (27), and others have been reported. Therefore, high-mannose glycans on the viral surface are attractive targets for the development of broadly effective antiviral agents. Alternatively, the levels of high-mannose glycans on the host cell surface increase upon influenza virus infection, leading to the activation of inflammatory responses upon recognition by innate immune mannan-binding lectins (28).

All OAA homologs contain a sequence repeat of ∼66 amino acids, with the number of repeats varying among different family members. The founding member OAA and *Pseudomonas fluorescens* lectin, designated as PFL, contain two tandem sequence repeats, whereas agglutinins from *Myxococcus xanthus* and *Burkholderia oklahomensis* contain four tandem sequence repeats (18, 19, 29, 30). The epitope of high-mannose glycans was identified as 3α,6α-mannopentaose (Manα(1–3)[Manα(1–3)[Manα(1–6)]Manα(1–6)]Man), the branched core unit of Man-9, a unique recognition element for OAA-family lectins (18).

To date, the crystal structures of OAA, PFL, and agglutinins from *M. xanthus* and *B. oklahomensis* are known (30, 31, 32, 33). All these structures reveal a β-barrel-like domain consisting of two sequence repeats and two symmetrically positioned glycan-binding sites at the opposite ends of the barrel. The OAA protein slightly changes its conformation upon binding to glycan (31, 32). Such conformational change has been proposed to be due to a “population shift” mechanism rather than to the common “induced fit” mechanism (34).

Here, we determined the X-ray crystal structures of the free and glycan-bound forms of *Pseudomonas taiwanensis* lectin (PTL), one of the OAA-family lectins consisting of two sequence repeats, the most potent among the three *Pseudomonas* genus-derived lectins (PFL, PTL, and *Pseudomonas mandelii* lectin) (17). Similar to other OAA-family lectins, PTL has a β-barrel-like structure with two symmetrically positioned glycan-binding sites at the opposite ends of the barrel. When comparing the crystal structures of PTL in free and glycan-bound forms, the conformation of the protein seems to change to a greater degree upon binding of mannopentaose than expected from the structural analysis of OAA (31, 32, 34). The electron density of the bound glycans also indicated that the binding affinities are different at the two glycan-binding sites, although the two sites display almost identical structures.

We then constructed site-specific mutated lectins based on structural insights from X-ray crystallography. Amino acid residues that surround the direct ligand-interacting residues in the weaker glycan-binding pocket were replaced with other amino acid residues by referring to the stronger glycan-binding pocket. Such mutations were estimated to boost the lectin population adopting a suitable conformation for glycan binding, and some mutated lectins exhibited enhanced antiviral activity. We propose that site-specific mutagenesis based on structural insights is an effective strategy for the generation of more powerful antiviral lectins.

## Results

### Crystal structures of PTL

**Figure 1:**
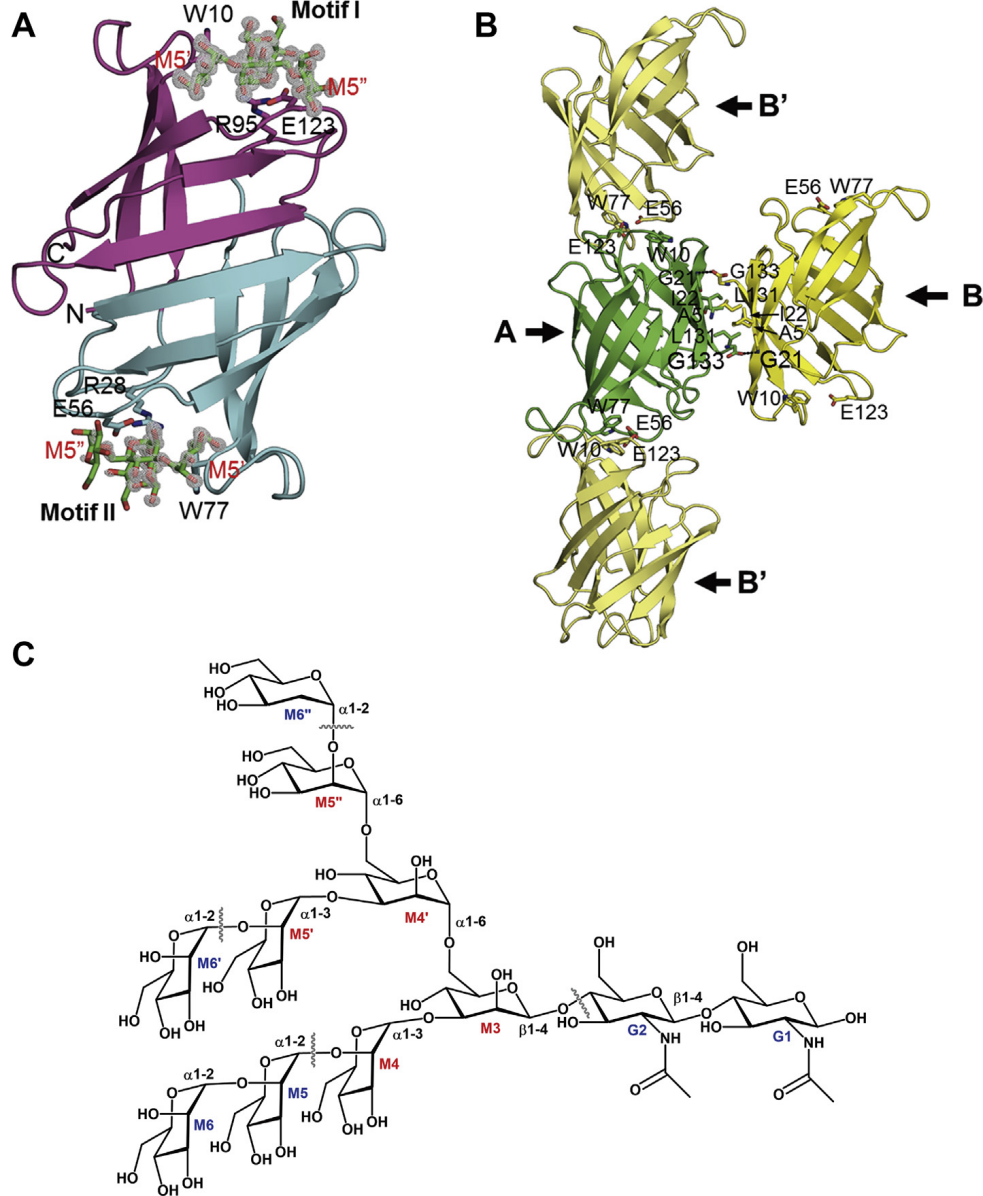
**Crystal structure of PTL.***A*, ribbon diagram of glycan-bound PTL Motifs I and II are shown in *magenta* and *cyan*, respectively. The bound glycan molecules are shown in the *stick model*. The *F*_o_-*F*_c_ electron density map, which was computed after removal of the glycans, is contoured at 2.5 σ. *B*, intermolecular interactions observed in the crystal of the free form of PTL. Monomers A (*light green*) and B (*gold*) generate a dimer-like structure in an asymmetric unit, while a fiber-like structure is generated *via* interaction between monomer A and monomer B′, which is related to monomer B by crystallographic symmetry. *C*, chemical structure of high-mannose glycan. G and M mean *N*-acetylglucosamine and mannose, respectively. Core structure inside the wavy lines is 3α,6α-mannopentaose used in the present study.

We determined the X-ray crystal structure of the free and glycan-bound forms of PTL (Table 1), an OAA-family lectin consisting of two tandem sequence repeats (18). Although the structure of the free form was determined at a moderately high resolution, the glycan-bound form was determined at an atomic resolution (0.95 Å), leading to very high model accuracy. The amino acid sequence of PTL is highly similar to those of OAA and PFL, whose three-dimensional structures have been previously determined (31, 32, 33). Similar to OAA and PFL, the X-ray crystal structures of both free and glycan-bound PTL reveal a β-barrel-like structure consisting of ten β-strands, with two symmetrically positioned glycan-binding sites at the opposite ends of the barrel (Fig. 1*A*). The PTL protein consists of motif I, comprising G94–G133 and M1–G26 residues, and motif II, including G27–G93 residues. If the *C*-terminal G133 residue was linked to the *N*-terminal M1 residue *via* one amino acid residue, the motifs I and II of PTL would be more similar than those of the resolved PTL structure.

**Table 1:** Data collection and refinement statistics

Data set	Glycan-free PTL	Glycan-bound PTL
Data collection		
Beamline	BL44XU, SPring-8	BL26B1, SPring-8
Detector	EIGER16M	EIGER4M
Space group	*P*2_1_2_1_2_1_	*P*2_1_
Cell dimensions	*a* = 44.13 Å, *b* = 44.15 Å, *c* = 134.82 Å	*a* = 43.06 Å, *b* = 57.86 Å, *c* = 58.90 Å, *β* = 96.95°
Wavelength (Å)	0.9000	0.8500
Resolution (Å)	50.00–1.88 (1.98–1.88)	50.00–0.95 (0.97–0.95)
Total no. of reflections	127,644 (19,308)	599,075 (28,340)
Unique reflections	22,280 (3203)	180,042 (8818)
Redundancy^a^	5.7 (6.0)	3.3 (3.2)
Completeness (%)^a^	100.0 (100.0)	100.0 (99.9)
*R*_merge_ (%)^a^^,^^b^	16.1 (55.5)	5.0 (49.9)
*I*/σ^a^	8.9 (4.3)	11.1 (2.2)
CC(1/2)	0.989 (0.885)	0.994 (0.752)
Refinement		
Resolution (Å)	19.81–1.88 (1.97–1.88)	34.38–0.95 (0.96–0.95)
Used reflections	22,190 (2727)	179,955 (5942)
No. of atoms		
Protein	2014	2072
Ligand	-	224
Water/ion	241/25	526/20
*R* (%)	17.86 (20.22)	13.57 (19.63)
*R*_free_ (%)	21.68 (25.19)	14.77 (20.77)
Rms deviations^c^		
Bond length (Å)	0.006	0.004
Bond angle (°)	0.823	0.831
Mean *B*-factor (Å^2^)		
Protein	11.4	9.0
Ligand	-	13.9
Water/ion	22.8/42.7	28.1/38.0
Ramachandran plot		
Favored (%)	95.80	98.09
Allowed (%)	3.82	1.91
Disfavored (%)	0.38	0.00
PDB code	7DC0	7DC4

a Values in parentheses are for the highest resolution bin.

b *R*_merge_ = Σ|*I*-<*I*>|/Σ*I*, where *I* is the observed intensity and <*I*> is the mean value of *I*.

c Rms deviations were calculated using PHENIX (42).

Motif I builds a glycan-binding site containing a W10 residue (the W10 site), while motif II builds another glycan-binding site containing a W77 residue (the W77 site). Notably, the electron density of the bound glycan at the W10 site is stronger than that at the W77 site (Fig. 1*A*). Simultaneous refinement of occupancy and *B*-factor of individual atoms is challenging due to the strong correlation between them. When the occupancies of the atoms in the bound glycan were set to 1, the averaged equivalent *B*-factor of the atoms at the W77 site was calculated to be 16.4 Å^2^, approximately 1.4-fold higher than that at the W10 site (11.4 Å^2^), suggesting that the binding affinity at the W10 site is stronger than at the W77 site. Concerning each sugar residue in the mannopentaose glycan (Fig. 1*C*), differences in the averaged *B*-factors are 3.1 Å^2^ in M3, 6.4 Å^2^ in M4, 4.6 Å^2^ in M4’, 2.9 Å^2^ in M5’, and 16.3 Å^2^ in M5’. Atoms in M4 and M5’ residues have high *B*-factors due to the absence of direct interactions with PTL residues. The flexibility seems amplified at the W77 site.

**Figure 2:**
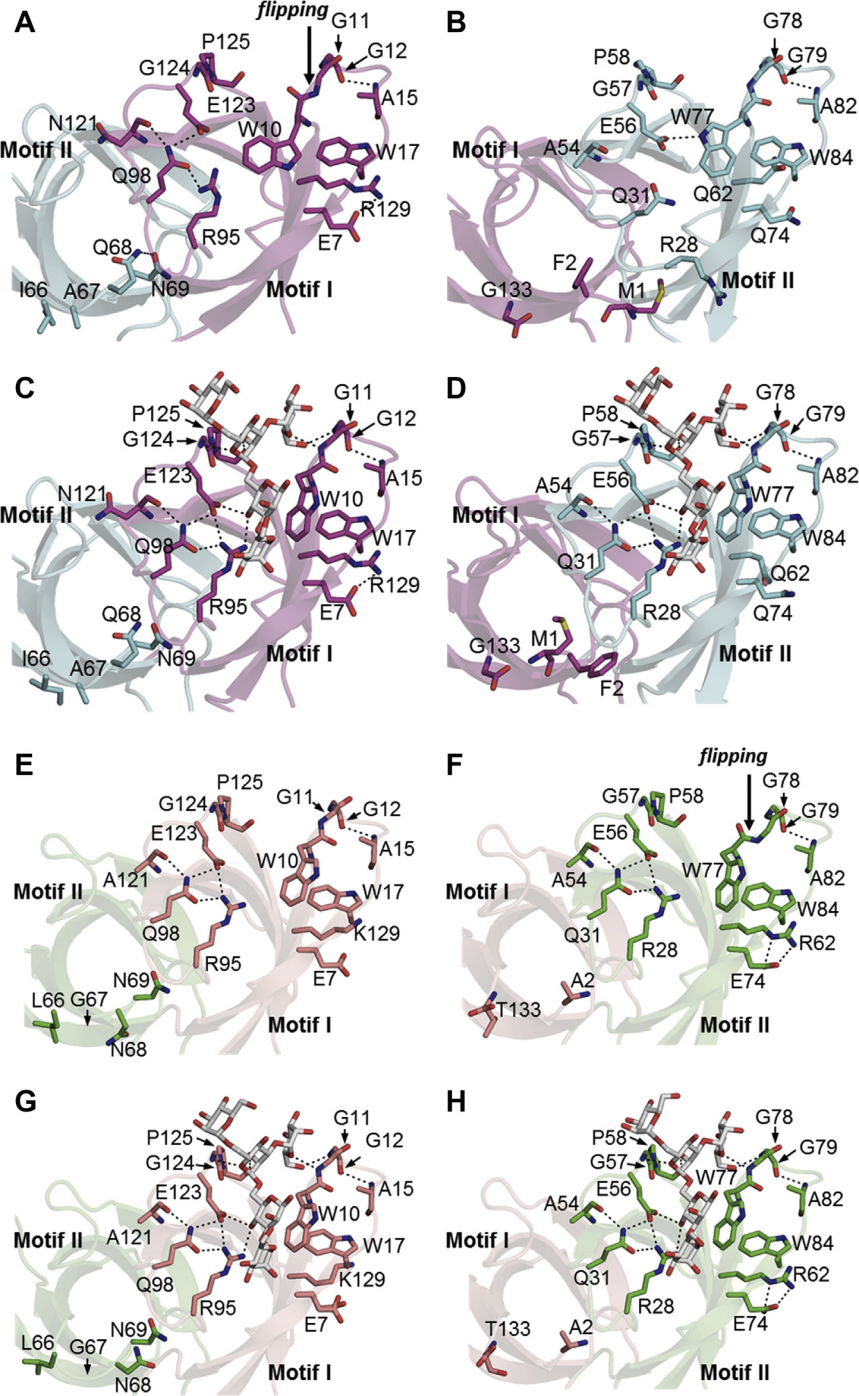
**Structure surrounding the glycan-binding site.***A*, W10 site in the free form of PTL, *B*, W77 site in the free form of PTL, *C*, W10 site in the glycan-bound form of PTL, *D*, W77 site in the glycan-bound form of PTL, *E*, W10 site in the free form of OAA, *F*, W77 site in the free form of OAA, *G*, W10 site in the glycan-bound form of OAA, *H*, W77 site in the glycan-bound form of OAA. Hydrogen bonds are shown as *dashed lines*. Motifs I and II of PTL are shown in *magenta* and *cyan*, respectively, while motifs I and II of OAA are shown in *salmon pink* and *lime green*, respectively.

**Figure 3:**
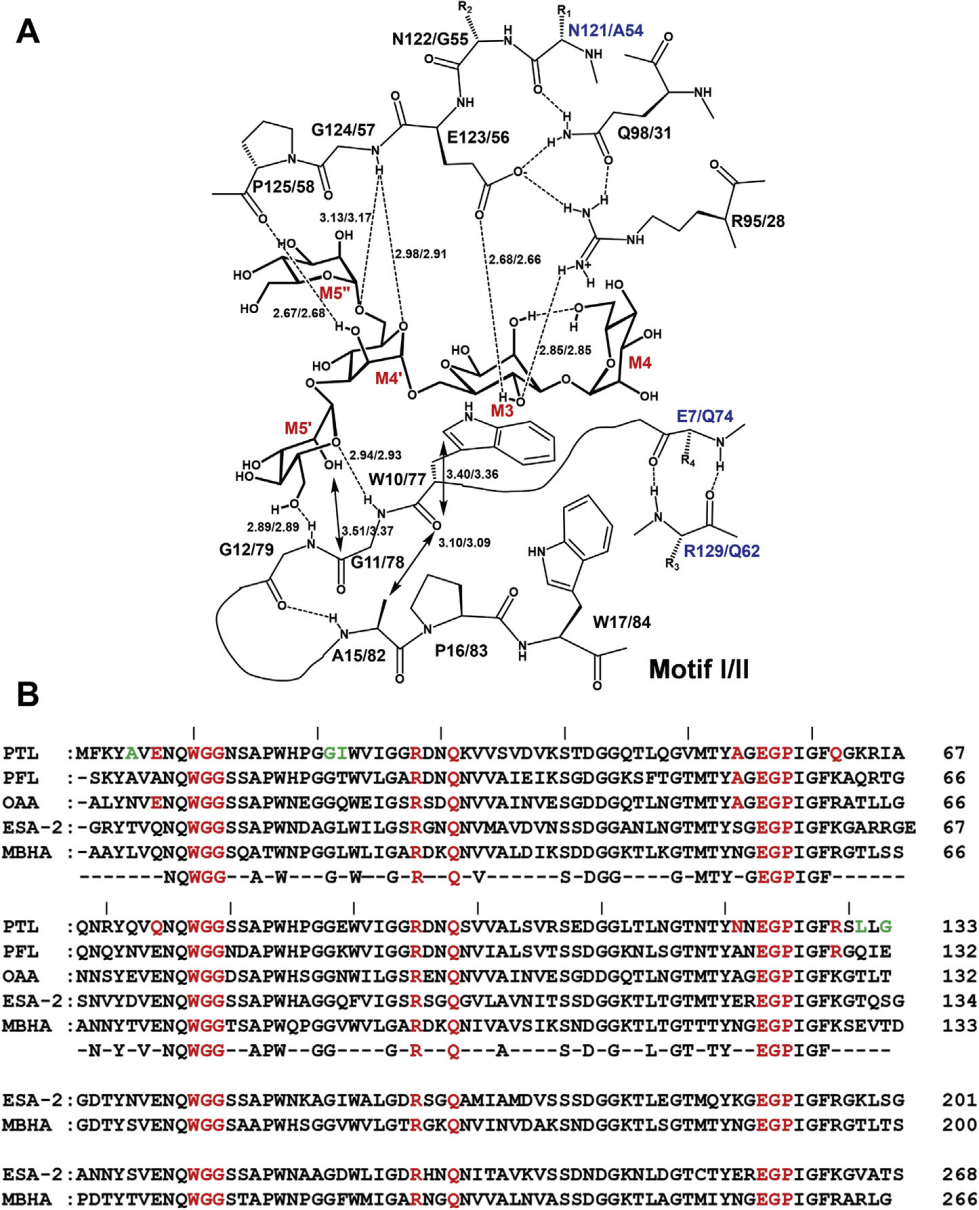
**Important residues for the binding to glycan in PTL.***A*, schematic diagram of the binding mode in the glycan-bound PTL. Hydrogen bonds and steric clashes are shown by *dotted lines* and *two-way arrows*, respectively. Distances between nonhydrogen atoms are shown in Å. Residues in mannopentaose are shown in *red*, while mutated residues in motif II of PTL and the counterparts in motif I are shown in *blue*. *B*, amino acid sequence alignment of OAA lectin family proteins. PTL, PFL, and OAA are displayed as two sequence repeats, while ESA-2 and MBHA are displayed as four sequence repeats, and the consensus residues are shown below. W10, W77, and their relevant surrounding residues are colored in *red*. L131 and their relevant surrounding residues are colored in *green*. ESA-2, *Eucheuma serra* lectin (268 aa); MBHA, *Myxococcus xanthus* lectin (266 aa); OAA, *Oscillatoria agardhii* lectin (132 aa); PFL, *Pseudomonas fluorescens* Pf0-1 lectin (132 aa); PTL, *Pseudomonas taiwanensis* lectin (133 aa).

In motif I, the side chains of the W10, R95, and E123 residues, the amide nitrogen atoms of the G124, G11, and G12 residues, and the carbonyl oxygen atom of the P125 residue contribute to glycan binding (Figs. 2*C* and 3*A*). Similarly, in motif II, the side chains of the W77, R28, and E56 residues, the amide nitrogen atoms of the G57, G78, and G79 residues, and the carbonyl oxygen atom of the P58 residue participate in glycan binding (Figs. 2*D* and 3*A*). These amino acid residues are highly conserved among OAA-family lectins (Fig. 3*B*), which bind a glycan molecule at both the W10 and W77 sites. It is important to note that in the glycan-bound form, the Cδ1 atom in the indole ring of the W10 or W77 residue is close to the carbonyl oxygen atom of the respective residue (Figs. 2, *C* and *D* and 3*A*), suggesting a conformational strain in the W10 and W77 residues.

In the crystals of both free and glycan-bound forms of PTL, one asymmetric unit contains two monomers. In the free form, the carboxyl group of the *C*-terminal G133 residue of one monomer interacts with the amide nitrogen atom of the G21 residue of the other monomer, leading to the generation of a dimer-like structure, related by a noncrystallographic twofold axis crossing near the A5, I22, and L131 residues (Fig. 1*B*). The formation of the dimer buries an accessible surface area of approximately 390 Å^2^ for each monomer, corresponding to approximately 20% of the total monomer surface area. This is a relatively small area compared with the values observed for other protein dimers, which ranged from 368 to 4746 Å^2^ (35), indicating a weak association of the monomers in the PTL dimer. The existence of a PTL dimer was not detected, except in the crystal structure (data not shown). Moreover, the other lectins, OAA and PFL, were not reported to form dimers, even in their crystal structures (31, 32, 33). We concluded that the formation of dimers in the crystal likely resulted from the crystallization process and that PTL exists predominantly as a monomer under physiological conditions.

The W10 site of one monomer was found to be in close contact with the W77 site of the adjacent monomer in the crystal, related to the other monomer by crystallographic symmetry (Fig. 1*B*). Consequently, fiber-like structures are assembled in the crystal of the glycan-free PTL. The formation of such fiber-like structures buries an accessible surface area of approximately 300 Å^2^ for each monomer, corresponding to approximately 15% of the total monomer surface area. Similar to the FimH lectin from *Escherichia coli* (36), the protein–protein interaction in PTL may block the binding of glycan, although biochemical evidence was not obtained. A dimer-like structure is also observed in the glycan-bound form of PTL. However, unlike in the free form, the W10 site is located far from the W77 site of the adjacent monomer in the glycan-bound form; thus, fiber-like structures are not assembled.

Although the two motifs in PTL display similar structures in the presence of glycan, their structures differ significantly in its absence. Indeed, the root mean square distance (rmsd) values after the superimposition of the two motifs from the free and glycan-bound forms were calculated to be 0.90 and 0.42 Å for 65 Cα atoms, respectively (Table 2). In particular, motif II in the free form adopts a different structure from that of motifs I and II in the glycan-bound form, with rmsd values of 0.90 and 0.82 Å for 65 Cα atoms, respectively. In the crystal structure of OAA (31, 32), the two motifs are more similar to each other than the case of PTL. The superimposition of motifs I and II in the free and glycan-bound forms of OAA yields rmsd values of 0.67 and 0.41 Å for 65 Cα atoms, respectively (Table 2).

**Table 2:** Pairwise rmsd values for each pair of the motifs in PTL and OAA^a^

		Glycan-free PTL		Glycan-bound PTL		Glycan-free OAA		Glycan-bound OAA	
		Motif I	Motif II	Motif I	Motif II	Motif I	Motif II	Motif I	Motif II
Glycan-free PTL	Motif I	0	0.903	0.489	0.533	0.600	0.573	0.682	0.561
	Motif II	-	0	0.903	0.820	1.01	0.966	1.06	0.912
Glycan-bound PTL	Motif I	-	-	0	0.416	0.478	0.614	0.523	0.337
	Motif II	-	-	-	0	0.524	0.604	0.565	0.385
Glycan-free OAA	Motif I	-	-	-	-	0	0.673	0.287	0.421
	Motif II	-	-	-	-	-	0	0.713	0.574
Glycan-bound OAA	Motif I	-	-	-	-	-	-	0	0.401
	Motif II	-	-	-	-	-	-	-	0

a Residues 2 to 26 and 94 to 133 in motif I and residues 27 to 66 and 69 to 93 in motif II were used for the calculations. Values are in Å.

In the case of OAA (31, 32), the structures surrounding each of the W10 and W77 sites are similar in the presence or absence of glycan, except for a peptide-bond flip between the W77 and G78 residues in the free form (Fig. 2, *E*–*H*). Flipping results in the inability to bind glycan at the W77 site, since a hydrogen bond cannot be formed between the G78 residue in OAA and the M5’ residue in a glycan molecule. Alternatively, PTL structures around both the W10 and W77 sites are modified upon glycan binding (Fig. 2, *A*–*D*). The structures surrounding the W10 and W77 sites of PTL are similar in the glycan-bound form (Fig. 2, *C* and *D*), however, significantly different from the corresponding structures in the free form (Fig. 2, *A* and *B*). Structural differences between the two glycan-binding sites found in the free form are partially caused by the crystallographic packing effect. As described above, the W10 site is close to the W77 site of the adjacent monomer (Fig. 1*B*), which may lead to the deformation of each glycan-binding site. Additionally, the W10 and W77 residues are in close contact with the E56 and E123 residues of the adjacent monomers, respectively (Fig. 1*B*). Accordingly, as the E56 residue is positioned near the E123 residue of the adjacent monomer, the electrostatic repulsion may be generated between the negatively charged residues. Intermolecular interactions may not occur in solution.

Unlike in OAA, the side chains of W10 and W77 residues in the free form of PTL occupy the glycan-binding sites, thereby possibly interfering with glycan binding (Fig. 2, *A* and *B*). Intermolecular contact may contribute to this orientation (Fig. 1*B*). Concerning the main chain, the orientation of the peptide bond between W77 and G78 residues in PTL is unchanged in the presence or absence of glycan (Fig. 2, *B* and *D*). However, in the glycan-free form of PTL, the W77 residue displays a lower conformational strain than in the glycan-bound form, as the change in the orientation of the indole ring moves the Cδ1 atom away from the carbonyl oxygen atom (Fig. 2*B*). Contrastingly, the orientation of the peptide bond between W10 and G11 residues in PTL is flipped by approximately 180° in the absence of glycan (Fig. 2, *A* and *C*). In this case, the W10 residue in the free form possesses a low conformational strain due to the great distance between the Cδ1 and carbonyl oxygen atoms, caused by the flipping of the peptide bond and/or change in the ring orientation (Fig. 2*A*). Therefore, the W10 and W77 residues at the two binding sites in the free form of PTL likely adopt more stable conformations than those in the glycan-bound form. Near the W10/W77 residues, conserved W17/W84 residues are present (Figs. 2, *A*–*D* and 3*A*). As described below, the tendency to flip the peptide bond may be changed by the residues interacting with the indole ring of W17/W84 (R129 and E7 at the W10 site and Q62 and Q74 at the W77 site).

At the W10/W77 site in the glycan-bound form of PTL, an extended hydrogen-bonding network is formed together with the bound glycan (Figs. 2, *C* and *D*, and 3*A*). The side chains of Q98/Q31 residues form hydrogen-bonding interactions with the carbonyl oxygen atom of the N121/A54 residues and the side chains of the E123/E56 and R95/R28 residues, respectively. The side chains of the E123/E56 residues are linked to those of the R95/R28 residues by a direct salt bridge and *via* a hydroxyl group in the bound glycan. This hydrogen-bonding network is partially maintained at the W10 site in the free form of PTL, although direct salt bridge is not found between the E123 and R95 residues (Fig. 2*A*). Alternatively, at the W77 site in the free form, the hydrogen-bonding network is destroyed (Fig. 2*B*). In this case, the Q31 residue does not form hydrogen-bonding interactions. The side chain of the R28 residue is oriented toward the outside of the glycan-binding pocket. However, the side chain of the E56 residue generates a new hydrogen bond with that of the W77 residue, possibly leading to stabilization of the local structure by compensating for the loss of hydrogen-bonding interactions.

### Site-specific engineered PTLs and their antiviral activity

Based on the above structural insights, we explored the relationship between the structure and function of PTL. We expected that site-specific engineering of the carbohydrate recognition site on the lectin protein would generate a new derivative with enhanced antiviral activity.

**Figure 4:**
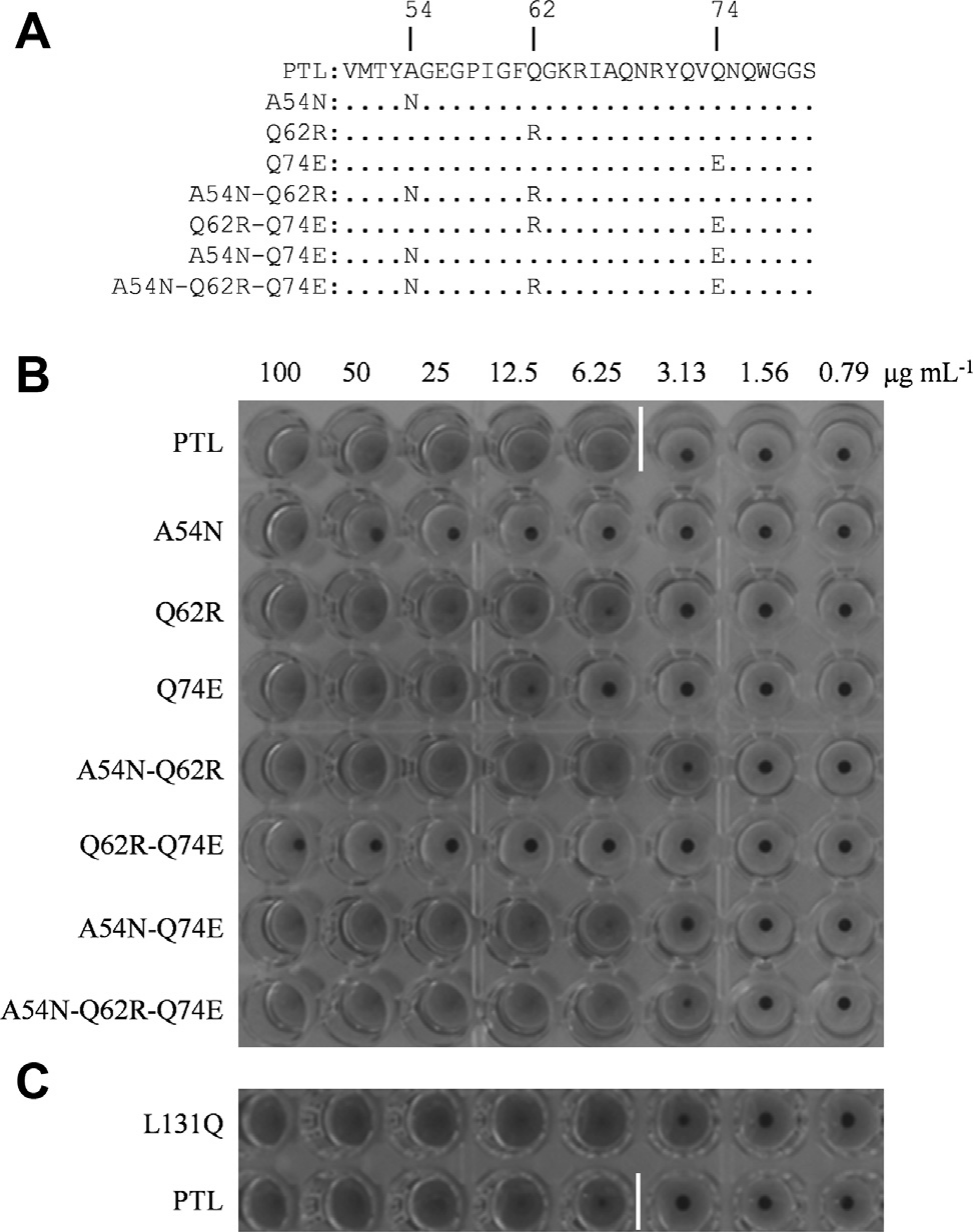
**Hemagglutination activity of mutated derivatives of PTL.***A*, site-directed mutated derivatives of PTL. The amino acids at positions 50 to 80 (motif II) of the seven site-directed mutated derivatives of PTL are shown. PTL derivatives with one point mutation are termed A54N, Q62R, and Q74E; those with two point mutations are termed A54N-Q62R, Q62R-Q74E, and A54N-Q74R; and that with three point mutations is termed A54N-Q62R-Q74E. These derivatives were expressed in *E. coli* as described in the Experimental procedures. Hemagglutination activity of purified PTL and its site-mutated derivatives. *B*, seven site-directed mutated derivatives or *C*, the L131Q derivative were incubated with rabbit erythrocytes. PTL exhibited hemagglutination up to 6.25 μg ml^−1^, as shown by *white vertical bar*.

**Figure 5:**
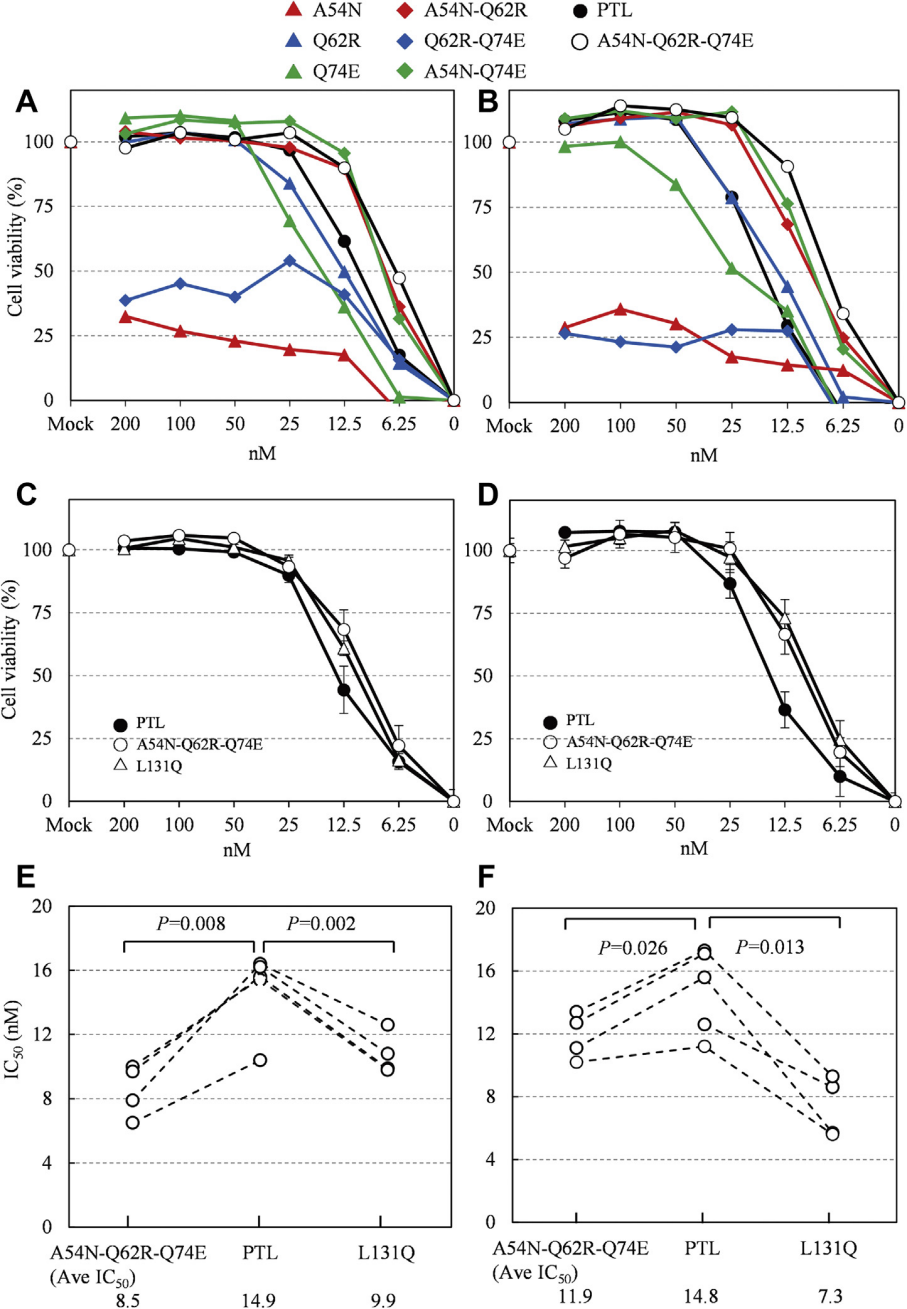
**Anti-influenza virus activity of mutated derivatives of PTL.***A* and *B*, anti-influenza virus activity of PTL and its derivatives mutated at the binding site in motif II. NCI-H292 cells were infected with the influenza virus strain (*A*) A/H1N1Oita/OU1P3-3/09 or (*B*) A/H3N2/Udorn/72 in the presence or absence of serially diluted lectin solutions. At 24 h postinfection, the infected cell cultures were fixed with 80% acetone and stained with amido black 10 B. Cell viability of cultures with each lectin concentration was plotted as a percentage of that in the mock culture (control). Each data point was obtained from the average of at least four measurements. The 50% lines were used to extract the IC_50_ of each type of lectin. Error bars are omitted for clarity. *C* and *D*, anti-influenza virus activity of PTL and its L131Q derivative. NCI-H292 cells were infected with influenza virus strains (*C*) A/H1N1Oita/OU1P3-3/09 or (*D*) A/H3N2/Udorn/72 in the presence or absence of serially diluted lectin solutions. Each data point was obtained from the average of at least four measurements. Values are shown as mean ± SEM. *E* and *F*, comparison of IC_50_ between PTL and its derivatives A54N-Q62R-Q74E and L131Q in (*E*) A/H1N1Oita/OU1P3-3/09 or (*F*) A/H3N2/Udorn/72. Antiviral potency was determined as the concentration of the inhibitor required for 50% inhibition of infection (IC_50_). IC_50_ values were plotted with four independent experiments. Statistical analysis was performed using a two-tailed paired Student’s *t*-test.

Amino acid residues that surround directly interacting residues in the weaker glycan-binding pocket (*i.e.*, around the W77 site in motif II) were replaced with other amino acid residues by referring to the stronger glycan-binding pocket (*i.e.*, around the W10 site in motif I). We focused on the three residues A54, Q62, and Q74 at the weaker-affinity W77 site, corresponding to the N121, R129, and E7 residues at the stronger-affinity W10 site, respectively (Figs. 2, *C* and *D*, and 3*A*). In total, seven mutated lectins were constructed with one, two, or three-point mutations at the codons of these three residues (Fig. 4*A*). These seven mutated PTLs were examined for their biological functions, that is, for their hemagglutination activity against rabbit erythrocytes (Fig. 4*B*) and antiviral activity against the influenza virus (Fig. 5, *A* and *B*).

The mutated lectins exhibited different hemagglutination activities. In particular, A54N and Q62R-Q74E derivatives displayed drastically lower hemagglutination activity, whereas the Q74E derivative showed a modest decrease compared with the parental PTL. Conversely, A54N-Q62R and A54N-Q62R-Q74E derivatives exhibited slightly enhanced hemagglutination activity. Finally, the other derivatives (Q62R and A54N-Q74E) showed activity similar to that of the parental PTL.

OAA-family lectins, including PTL, bind high-mannose glycans on the HA protein of the influenza virus, thereby inhibiting the entry of viral particles. Concerning their anti-influenza activity, the inhibitory potential of each derivative was highly correlated with the respective hemagglutination activity. The A54N and Q62R-Q74E derivatives showed diminished antiviral activity, whereas the activity of the Q74E derivative was slightly lower than that of the parental PTL. Finally, A54N-Q62R, A54N-Q74E, and A54N-Q62R-Q74E derivatives retained their ability to inhibit the entry of the influenza virus into host cells with a similar or higher potency than that of the parental PTL. These results indicate that structural changes or population shifts caused by site-specific amino acid replacement led to changes in the binding ability of lectins to erythrocytes and viral particles.

In particular, A54N and Q62R-Q74E mutations resulted in a drastic decrease in hemagglutination activity and antiviral activity, indicating that these amino acid replacements disrupted the conformation suitable for glycan binding around the W77 site. However, the A54N-Q62R-Q74E triple mutation restored the binding activity of lectin, suppressing the negative effects of A54N or Q62R-Q74E mutations. The effect of replacement at these three residues may depend on whether the modified conformation favors glycan binding.

The L131 residue seems to participate in the dimerization of PTL through hydrophobic interactions with the L131 residue of the other monomer (Fig. 1*B*). Such dimerization seems to contribute to the condensation of the glycan-binding sites, as observed in agglutinins from *M. xanthus* and *B. oklahomensis*, containing four sequence repeats (30, 33). Since the Q131 residue of PFL, which does not generate a dimer-like structure in the crystal (33), corresponding to L131 of PTL (Fig. 4*B*), it is of interest to determine whether the replacement of L131 affected the binding activity of this lectin. Therefore, another type of engineered lectin, L131Q, was constructed to examine its hemagglutination and anti-influenza activities. As shown in Figures 4*C* and 5, *C*–*F*, the hemagglutination and anti-influenza activities of this mutated variant were modest, though significantly enhanced compared with those of the parental PTL.

**Figure 6:**
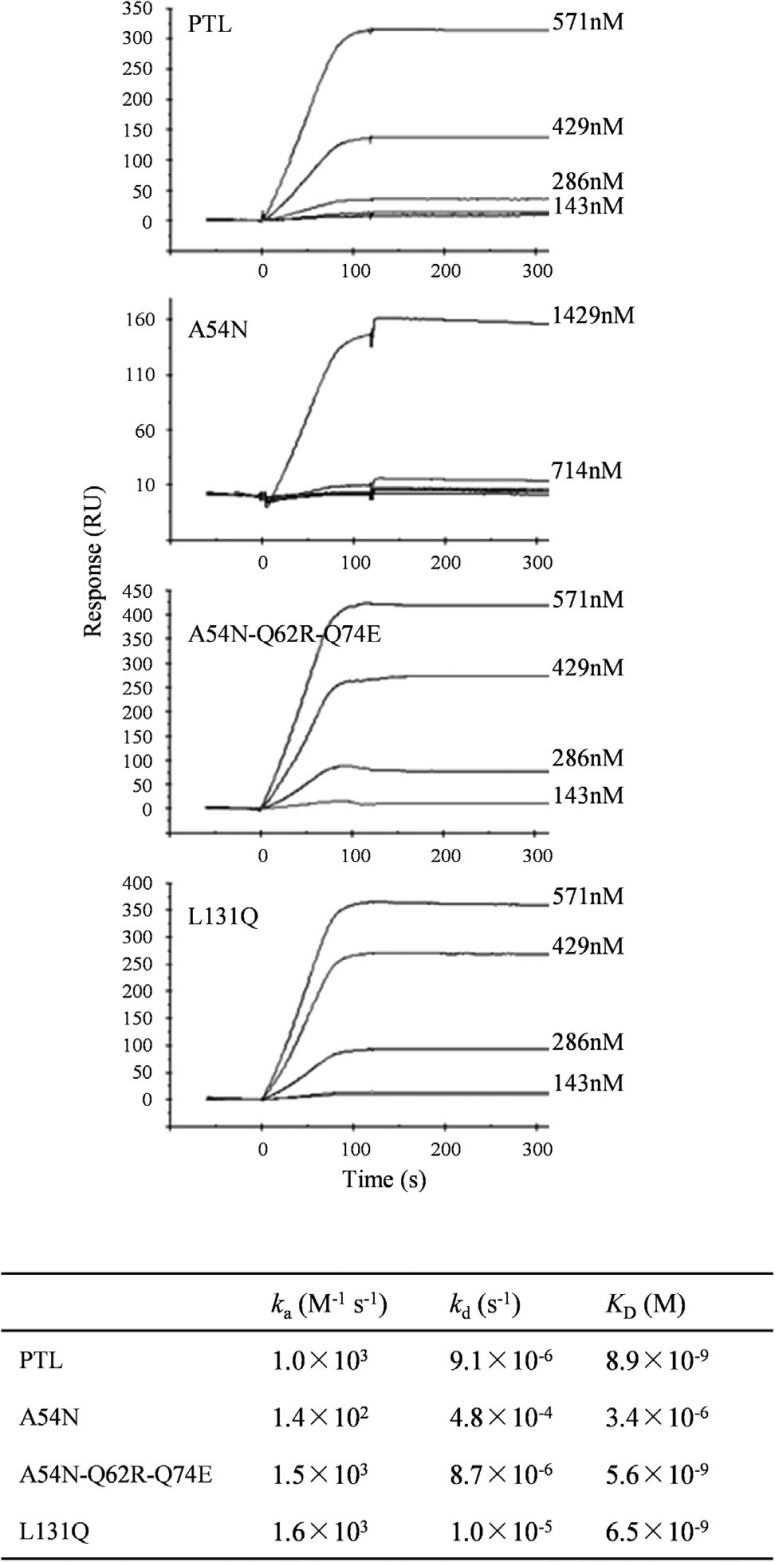
**Interaction of PTL and its derivatives to influenza virus HA protein.** Direct interaction of PTL and its derivatives (A54N, A54N-Q62R-Q74E, and L131Q) to the HA protein was analyzed by SPR. Following the immobilization of the HA ligand onto the CM5 sensor chip, lectin solution at the indicated concentrations was injected. Sensorgrams for the binding were recorded in real time, and the response is expressed in resonance units (RU). Binding kinetics of the interaction between lectin and the ligand are shown. *k*_a_, association rate constant, *k*_d_, dissociation rate constant, *K*_D_, dissociation constant.

The binding affinity of the parental PTL and the three mutants (A54N, A54N-Q62R-Q74E, and L131Q) to the influenza HA proteins was investigated by surface plasmon resonance (SPR) analysis (Fig. 6). The results were consistent with the biological activities. A high-aﬃnity interaction to the HA protein, which was deduced from the dissociation constants in the nanomolar range, was detected in the parental PTL, the A54N-Q62R-Q74E, and L131Q mutants. Notably, the binding affinity of A54N-Q62R-Q74E and L131Q mutants was enhanced compared with that of the parental PTL. Alternatively, the affinity of the A54N mutant was three orders of magnitude lower than that of the parental PTL.

## Discussion

Previously, we analyzed the binding specificity of OAA toward various high-mannose glycans (18), suggesting that G2, M3, M4’, and M5’residues in the glycans (Fig. 1*C*) are essential for binding to the protein. Additionally, attachment of the M4 residue to the core was found to slightly increase the binding affinity, whereas attachment of the M6’ residue significantly reduced the affinity. The crystal structure, in which M3, M4’, and M5’ have intermolecular interactions with PTL, while M4 has an intramolecular hydrogen bond with M3 (Fig. 3*A*), agrees well with the previous result. Although the crystal structure does not imply the necessity of the G2 moiety, the orientation of the C1 hydroxyl group in M3 indicates that the mannose moiety is a β-anomer, suggesting that PTL selectively binds to a glycan containing a β-anomer at the M3 site from a mixture containing α- and β-anomers. If M3 was an α-anomer, a van der Waals repulsive force would be established between the C1 hydroxyl group and indole ring of W10/W77 (Fig. 3*A*), thus interfering with the binding to OAA and PTL. Previously (18), pyridylaminated glycans were used to analyze binding affinity toward OAA. When the C1 site of M3 was pyridylaminated, the cyclic structure at the M3 residue was destroyed, resulting in a weak binding affinity to OAA. Contrastingly, the C2 hydroxyl group of M5’ was close to the carbonyl carbon of G11/G78 (Fig. 3*A*). Therefore, the attachment of the M6’ moiety to the C2 hydroxyl group of M5’ may generate a steric crash on the binding to OAA or PTL, thus interfering with the binding to the lectins.

As described above, the motifs I and II of OAA, PFL, and PTL must have distinct structures, since these proteins are not cyclic peptides. In general, the formyl methionine residue encoded by the start codon ATG is removed from bacteria. However, the electron density map of PTL indicates the presence of the M1 residue. In the free form of PTL, the M1–F2 region residing in motif I is stabilized in a form different from that of the corresponding region (Q68–N69) in motif II. This may result in a conformational change in the R28–Q31 region, leading to a conformation that is largely different from that of the glycan-bound form (Fig. 2, *A* and *B*). Alternatively, the M1 residue of OAA and PFL is likely removed posttranslationally, and the two motifs in the free forms of OAA and PFL are structurally more similar than those of PTL (Fig. 2, *E* and *F*) (31, 32, 33). These observations suggest that the presence of the M1 residue may trigger a certain conformation around the W77 site of PTL, different from that of the glycan-bound form.

The structures around the W10 and W77 sites in OAA are similar in the presence or absence of glycan, except for a difference in the peptide bond between W77 and G78 residues (Fig. 2, *E*–*H*) (31, 32). Indeed, the orientation of this peptide bond is flipped by approximately 180° in the absence of glycan (Fig. 2, *F* and *H*). Such conformational changes prevent the formation of a hydrogen bond between the amide nitrogen atom of the G78 residue and the C6 hydroxyl group of the M5’ residue in the bound glycan (Fig. 2*H*). Moreover, around the W77 site of OAA, the side chain of the W84 residue displays a stacking interaction with the side chain of the R62 residue, which in turn forms a salt bridge with that of the E74 residue (Fig. 2, *F* and *H*). This stacking interaction may shift the β-turn region (G79–A82) toward the glycan-binding pocket. If the orientation of the peptide bond between the W77 and G78 residues was identical to that in the glycan-bound form, this movement of the β-turn would lead to the carbonyl oxygen atom of the W77 residue and the methyl group of the A82 residue to adopt a distance shorter than the van der Waals contact distance. Therefore, in the absence of glycan, the W77–G78 peptide bond must be flipped to avoid van der Waals repulsive forces (Fig. 2*F*). Additionally, flipping removes the conformational strain caused by the close contact between Cδ1 and the carbonyl oxygen atoms of the W77 residue (Fig. 2*F*). In contrast, the equivalent peptide bond at the W10 site (W10–G11) of OAA essentially remains unchanged in the presence or absence of glycan (Fig. 2, *E* and *G*), suggesting that this site adopts the bound conformation even without any glycan present. In this case, the corresponding β-turn region (G12–A15) does not move toward the glycan-binding pocket. Although this position of the β-turn may be a disadvantage for glycan binding, the conformational strain would still be too low to require flipping of the W10–G11 peptide bond.

Conversely, in the case of PTL, the orientation of the peptide bond between W10 and G11 residues is flipped in the absence of glycan (Fig. 2, *A* and *C*). The W10 site of PTL shows a strong structural similarity to the W77 site of OAA (Fig. 2, *A*, *C*, *F* and *H*). The side chain of the W17 residue of PTL displays a stacking interaction with the side chain of the R129 residue, which in turn forms a salt bridge with that of the E7 residue (Fig. 2, *A* and *C*). Such stacking interactions may shift the β-turn region (G12–A15) toward the glycan-binding pocket, and the W10–G11 peptide bond would be flipped in the free form to avoid the establishment of van der Waals repulsive forces between the carbonyl oxygen atom of the W10 residue and the methyl group of the A15 residue (Fig. 2*A*). Although the distance between the carbonyl oxygen atom of the W77 residue and the methyl group of the A82 residue is short in PTL (2.94 Å), the conformational strain would still be too low to require flipping of the W77–G78 peptide bond.

According to the “population shift” theory (34), there are two possible conformations for the glycan-binding site in the absence of glycan: the first is suitable for glycan binding despite a high conformational strain (conformation A), whereas the second hampers glycan binding due to the flipped peptide bond, however, shows a low conformational strain (conformation B). At the W10 site of PTL, the population adopting conformation A seems to be less abundant than adopting conformation B. However, the W10 site in conformation A could selectively bind glycan, thereby incrementally inducing a conformational change from B to A according to the chemical equilibrium. Such a population shift may continue until the exhaustion of unbound sites. Alternatively, the force shifting the β-turn region would generate a strong binding affinity toward glycan, consistent with the stronger electron density of glycan molecules observed at the W10 site (Fig. 1*A*). Although the electron densities of the bound glycans are different at the two binding sites, the binding modes of glycans to PTL are almost identical (Fig. 3*A*). However, the structure at the W77 site seems to be slightly less appropriate for glycan binding than that at the W10 site. For instance, the conformational strain between the C2 hydroxyl group of M5’ and the carbonyl carbon of G78 is high, whereas the hydrogen-bonding interaction between the C6 hydroxyl group of M4’ and the main-chain amide of G57 is weak.

The W17 and W84 residues are highly conserved among OAA-family lectins, whereas the R62 and E74 residues of OAA and the R129 and E7 residues of PTL are not (Fig. 3*B*). The presence of K129 and E7 residues around the W10 site of OAA fails to trigger flipping of the W10–G11 peptide bond in the absence of glycan (Fig. 2*E*) (31). Similarly, Q62 and Q74 residues are present around the W77 site of PTL, impeding the flipping of the W77–G78 peptide bond in the absence of glycan (Fig. 2*B*). In the free form of PFL (33), R129 and A7 residues are present around the W10 site, whereas K62 and E74 residues are present around the W77 site (Fig. 3*B*). In this type of lectin, the W10–G11 peptide bond is not flipped in the absence of glycan, whereas the W77–G78 peptide bond is flipped in one of the two monomers in the asymmetric unit of the crystal. These observations strongly indicate that flipping of the peptide bond is stimulated by the presence of a pair of R129/R62 and E7/E74 residues. Q62R-Q74E exhibited decreased antiviral activity in site-specific mutated PTLs. Such replacement may stimulate flipping of the peptide bond between the W77 and G78 residues in the absence of glycan, leading to a decrease in the population adopting a suitable conformation for glycan binding.

The A54 residue in motif II is common in PTL, PFL, and OAA, whereas the corresponding 121st residue in motif I is alanine in PFL and OAA, however asparagine in PTL (Fig. 3*B*). Among our site-specific mutated PTLs, A54N exhibited lower antiviral activity. Therefore, the introduced side chain of the asparagine residue may form a hydrogen-bonding interaction with the main-chain carbonyl oxygen atom of the same residue. This interaction inhibits the formation of a hydrogen bond between the main-chain carbonyl oxygen of the N54 residue and the side chain of the Q31 residue, which is a part of the hydrogen-bonding network formed in the glycan-bound form (Fig. 2*D*). As a result, the A54N replacement resulted in an increased population adopting a conformation different from that of the glycan-bound form near the Q31 residue, leading to a lower binding affinity of the W77 site toward glycan.

In PTL, motif I seems to have a stronger glycan-binding affinity than the original motif II. Therefore, since motif II in the A54N-Q62R-Q74E triple mutant of PTL most resembles motif I among those of all seven PTL mutants, the triple mutant was predicted to exhibit stronger glycan-binding potential than the parental PTL, leading to higher hemagglutination activity and antiviral activity. As expected, the A54N-Q62R-Q74E triple mutation in PTL led to higher antiviral activity (Fig. 5). In this case, the A54N replacement may promote a conformational change in the R28–Q31 region in the absence of glycan. The Q62R-Q74E replacement may trigger the flipping of the W77–G78 peptide bond. Both conformational changes are likely to decrease the binding affinity of PTL toward glycans. However, due to van der Waals repulsive forces between the flipped carbonyl oxygen and indole ring of W77, the possible conformations of the W77 residue are restricted. Therefore, simultaneous conformational changes in the R28–Q31 region and flipping of the W77–G78 peptide bond may not occur, since the hydrogen-bonding interaction between E56 and W77 residues, found in the crystal structure of the free form of PTL (Fig. 2*B*), would not be formed due to the conformational restriction on the W77 residue. As a result, the population adopting a suitable conformation for glycan binding might increase to some extent, leading to a higher binding affinity of PTL toward glycan.

In general, lectins usually possess multivalent binding sites, which enable their agglutination (37). The lectin inhibits viral entry into host cells by directly binding to viral surface glycoproteins, thereby exerting potent antiviral activity. Multivalent binding may not necessarily be required for antiviral activity. The parental PTL and the A54N-Q62R-Q74E mutant demonstrated strong binding affinities to influenza HA. Alternatively, the affinity of the A54N mutant to influenza HA was three orders of magnitude lower than that of parental PTL, possibly due to a reduced binding affinity in motif II caused by its mutation. The results suggest that the binding affinity is strongly influenced by divalent binding, in which two adjacent glycans protruding from HA proteins are intra- or inter-molecularly bridged by the divalent PTL. The divalent binding mode may greatly improve the apparent binding affinity toward viral proteins. Considering that the binding affinity of the A54N-Q62R-Q74E mutant, possibly displaying enhanced binding affinity in motif II, is only 1.6-fold higher than that of the parental PTL (Fig. 6), multivalency is more significant than the binding affinity at the individual binding site. The A54N and Q62R-Q74E mutants drastically decreased their hemagglutination and antiviral activities. Proper multivalency of PTL is likely important for robust antiviral activity.

When comparing the hemagglutination and antiviral activities between PTL and another mutant L131Q, the latter exhibited significantly higher activities than PTL (Figs. 4*C* and 5, *C*–*F*). Both PTL and the L131Q mutant possess identical motifs I and II. Dimer formation in PTL was barely detectable; however, a small number of dimers may exist. Although divalent binding seems to be necessary for strong antiviral activity, further condensation of the binding sites caused by dimer formation probably inhibits glycan binding.

We determined the crystal structures of PTL, an OAA-family lectin, to elucidate the structural basis of the glycan-binding mechanism and constructed several site-specific mutated PTLs based on these insights. To elucidate the structure–function relationship of PTL, the PTL derivatives were examined for their hemagglutination and anti-influenza virus activities. Interestingly, the derivatives A54N-Q62R-Q74E and L131Q exhibited higher antiviral activity than the parent PTL. Our findings suggest that genetic engineering strategies to increase the population of lectins adopting a suitable conformation for glycan binding is effective for creating more powerful microbicides with enhanced antiviral activity.

## Experimental procedures

### Viruses and cells

Viral stocks of influenza A/H1N1/Oita/OU1P3-3/09 and A/H3N2/Udorn/72 were used for viral infection. Viruses were grown in the chorioallantoic fluid of 10-day-old chicken eggs. Aliquots of each viral preparation were stored at −80 °C until use. Human lung carcinoma NCI-H292 cells (ATCC CRL1848) were used as host cells. The cells were grown in RPMI-1640 supplemented with 10% fetal bovine serum, 100 IU ml^−1^ penicillin, and 100 μg ml^−1^ streptomycin (Gibco).

### Crystallography

Prior to crystallization, the PTL solution was dialyzed against 20 mM Tris-HCl buffer (pH 8) containing (NaCl 0.1 M) and was then concentrated to 10 mg ml^−1^ using Amicon Ultra centrifugal filter units (Millipore). PTL crystals were grown using the sitting-drop vapor-diffusion method by mixing 2 μl of protein solution with 2 μl of precipitant solution. Stick-like crystals were formed within 3 days using 0.1 M Mes-NaOH buffer (pH 6.5) containing 1.5 M (NH_4_)_2_SO_4_ as the precipitant solution. The crystals were flash frozen before data collection with a cryoprotectant containing 30% (v/v) glycerol. The diffraction intensities of the crystals were collected using synchrotron radiation from BL44XU at SPring-8. X-ray diffraction was measured with a pixel detector equipped at the station, and the intensities were integrated using iMosflm (38), scaled, and merged using SCALA (39). The crystal structure of PTL was solved according to the molecular replacement method using the atomic coordinates of OAA (PDB code: 3S5V) as a search model and the program Molrep in the CCP4 program suite (40). The model was revised and refined using the programs COOT (41) and PHENIX (42). Each refinement cycle included the refinement of the positional parameters, individual isotropic *B*-factors, and revision of the model, which was visualized by the COOT program. To obtain crystals of glycan-bound PTL, 3α,6α-mannopentaose was purchased from Sigma-Aldrich and dissolved in a small amount of water. PTL and 3α,6α-mannopentaose were mixed at a molar ratio of 1:3 before crystallization using the sitting-drop vapor-diffusion method. Stick-like crystals were formed within 7 days using a 0.1 M Mes-NaOH buffer (pH 6.5) containing 30% (w/v) polyethylene glycol monomethyl ether 5000 and 0.2 M (NH_4_)_2_SO_4_ as precipitant solution. The diffraction intensities of the crystals were collected using synchrotron radiation from BL26B1 at SPring-8. The intensities were integrated using XDS (43), scaled, and merged using Aimless (44). For the glycan-bound PTL model, anisotropic *B*-factors were introduced for all atoms using PHENIX. A subset of 5% of the reflections was used to monitor the free *R* factor (*R*_free_) (45). Details of the data collection and refinement statistics are presented in Table 1. AREAIMOL (46) was used to calculate accessible surface area. The structures presented in this paper were shown in Supplemental Data S1 and S2.

### Cloning of site-directed mutated PTL cDNAs and protein purification

PTL cDNA (length: 402 bases; accession no. NZ_KE384450.1) was cloned into the pET101/D-TOPO vector and transformed into *the E. coli* K12 TOP10 strain using the Champion pET Directional TOPO Expression kit (Invitrogen) as previously described (17). To introduce the A54N point mutation on the PTL cDNA, the first half cDNA of mutated PTL was amplified using primers PTLstart5 and A54N3 with PTL cDNA as a template. In parallel, the second half of the cDNA of mutated PTL was amplified using primers A54N5 and PTLstop3 with PTL cDNA as a template. Then, the full-length A54N-mutated cDNA was amplified using primers PTLstar5 and PTLstop3 with the two half-mutated cDNAs as templates. Similarly, Q62R- and Q74E-mutated cDNAs were amplified. These A54N-, Q62R-, and Q74E-mutated cDNAs were cloned into the pET101/D-TOPO vector and transformed into the *E. coli* K12 TOP10 strain using the Champion pET Directional TOPO Expression Kit (Invitrogen). To construct A54N-Q62R and A54N-Q74E double-mutated cDNA, A54N cDNA was used as a template to introduce another Q62R mutation or Q74E mutation, respectively. To construct a Q62R-Q74E double-mutated cDNA, Q62R cDNA was used as a template to introduce another Q74E mutation. These three double-mutated cDNAs were cloned into the pET101/D-TOPO vector, as described above. To construct an A54N-Q62R-Q74E triple-mutated cDNA, A54N-Q62R cDNA was used as a template to introduce another Q74E mutation, and the triple-mutated cDNA was cloned into the pET101/D-TOPO vector as described above. To obtain these mutated cDNAs, PCR amplification was performed using the following primers: PTLstart5, 5′-CACCATGTTCAAGTACGCAGTGGA-3′ (start codon underlined); PTLstop3, 5′-CTAACCGAGCAGGCTGCGGAAG-3′ (stop codon underlined); A54N5, 5′-GGTGTCATGACCTATAACGGCGAGGGACCGA-3′ (mutated site underlined); A54N3, 5′-TCGGTCCCTCGCCGTTATAGGTCATGACACC-3′ (mutated site underlined); Q62R5, 5′-CCGATCGGCTTCAGGGGCAAGCGCATC-3′ (mutated site underlined); Q62R3, 5′-TGCGCTTGCCCCTGAAGCCGATCGGT-3′ (mutated site underlined); Q74E5, 5′-CCGCTACCAGGTGGAGAACCAGTGGGGT-3′ (mutated site underlined); and Q74E3, 5′-CCCCACTGGTTCTCCACCTGGTAGCGG-3′ (mutated site underlined). Finally, to construct an L131Q-mutated cDNA, PTL cDNA was used as a template to introduce a mutation at residue 131 using the primers PTLstart5 and L131Q3: 5′-CTAACCGAGCTGGCTGCGGAAG-3′ (stop codon and mutated site underlined). The presence of the desired site-directed mutations and the absence of any undesired mutations were confirmed in all the mutated sequences obtained by DNA sequencing.

For the expression and purification of the original PTL and PTL-derived lectins, the above plasmids were transformed into the *E. coli* K12 BL21 Star (DE3) strain (Invitrogen). The transformed *E. coli* cells were grown and the expression of these proteins was induced under the *lac* operon using 0.8 mM IPTG. After 6 h of incubation at 37 °C, *E. coli* cells were collected by centrifugation at 5800*g* for 20 min. Next, *E. coli* cells were suspended in phosphate buffered saline (20 mM phosphate buffer containing 0.15 M NaCl, pH 7.0) and then disrupted by sonication. The supernatants of the lysates were purified by loading on a Superose 12 column (GE Healthcare), as previously described (17).

### Hemagglutination activity

The purified PTL and its seven site-directed mutated derivatives were adjusted to an initial concentration of 100 μg ml^−1^. Fifty microliters of each lectin solution prepared by twofold serial dilution and 50 μl of 2% rabbit erythrocytes were added to each well of a 96-well plate, which was then agitated for 1 min. Hemagglutination was detected after incubation for 1 h at room temperature.

### Viral infection and its inhibition by lectins

NCI-H292 cells grown in 48-well plates were infected with influenza virus A/H1N1/Oita/OU1P3-3/09 or A/H3N2/Udorn/72 at a multiplicity of infection of 2.5. Lectins were added together with viral solutions to cell cultures. At 24 h postinfection, the infected cells were fixed with 80% acetone and stained with 0.5% amido black 10 B (Wako Pure Chemical Corporation) in 45% ethanol and 10% acetic acid. The stained plates were imaged on a gray scale. The color densities of the pictures were quantitated by densitometry using the NHI-Image J 1.48v software (Fig. S1). Infection of cell cultures in the absence of lectin resulted in severe cytopathic effects, leading to the almost complete disappearance of cells from the wells and to a cell viability of 0%. On the other hand, cells in the mock-infected cell cultures were intact and exhibited a cell viability of 100%, similar to previous reports (17).

### Surface plasmon resonance analysis

Direct interaction of the parental PTL and the three mutants (A54N, A54N-Q62R-Q74E, and L131Q) with influenza HA protein was assessed using a BIAcore X100 system. The CM5 sensor chips were activated with *N*-hydroxysuccinimide and *N*-ethyl-*N*’-dimethylaminopropyl carbodiimide. The influenza vaccine preparation (Denka-Seiken), which contains HA proteins mixture of A/California/7/09 (H1N1), A/Victoria/210/09 (H3N2), and B/Brisbane/60/08, was immobilized onto the sensor chip by a standard amine coupling method. The unreacted groups on the sensor surface were blocked with 1 M ethanolamine. Binding experiments were performed using various concentrations of PTL or the mutants at a flow rate of 30 μl min^−1^ with a running buffer consisting of 10 mM HEPES-NaOH (pH 7.4) and 150 mM NaCl. The following conditions were employed for the kinetics/affinity assay: contact time of 120 s and dissociation time of 600 s. The sensor surface was regenerated using 10 mM glycine-HCl (pH 1.5). Kinetic parameters were calculated using the Biacore X100 evaluation software (BIAcore International AB).

## Data availability

The structures presented in this paper have been deposited in the Protein Data Bank (PDB) (Supporting Information PDB ID: 7DC0, PDB ID: 7DC4).

## Supporting information

This article contains supporting information.

## Conflict of interest

The authors declare no conflicts of interest.
