# Supplementary material for: Lectins engineered to favor a glycan-binding conformation have enhanced antiviral activity
Source: J Biol Chem. 2021 Apr 23;296:100698. doi: 10.1016/j.jbc.2021.100698 (PMC8166773; doi:10.1016/j.jbc.2021.100698)
Supplement: Supplemental Figure S1 [file mmc3.pdf]

Supplementary Fig. S1

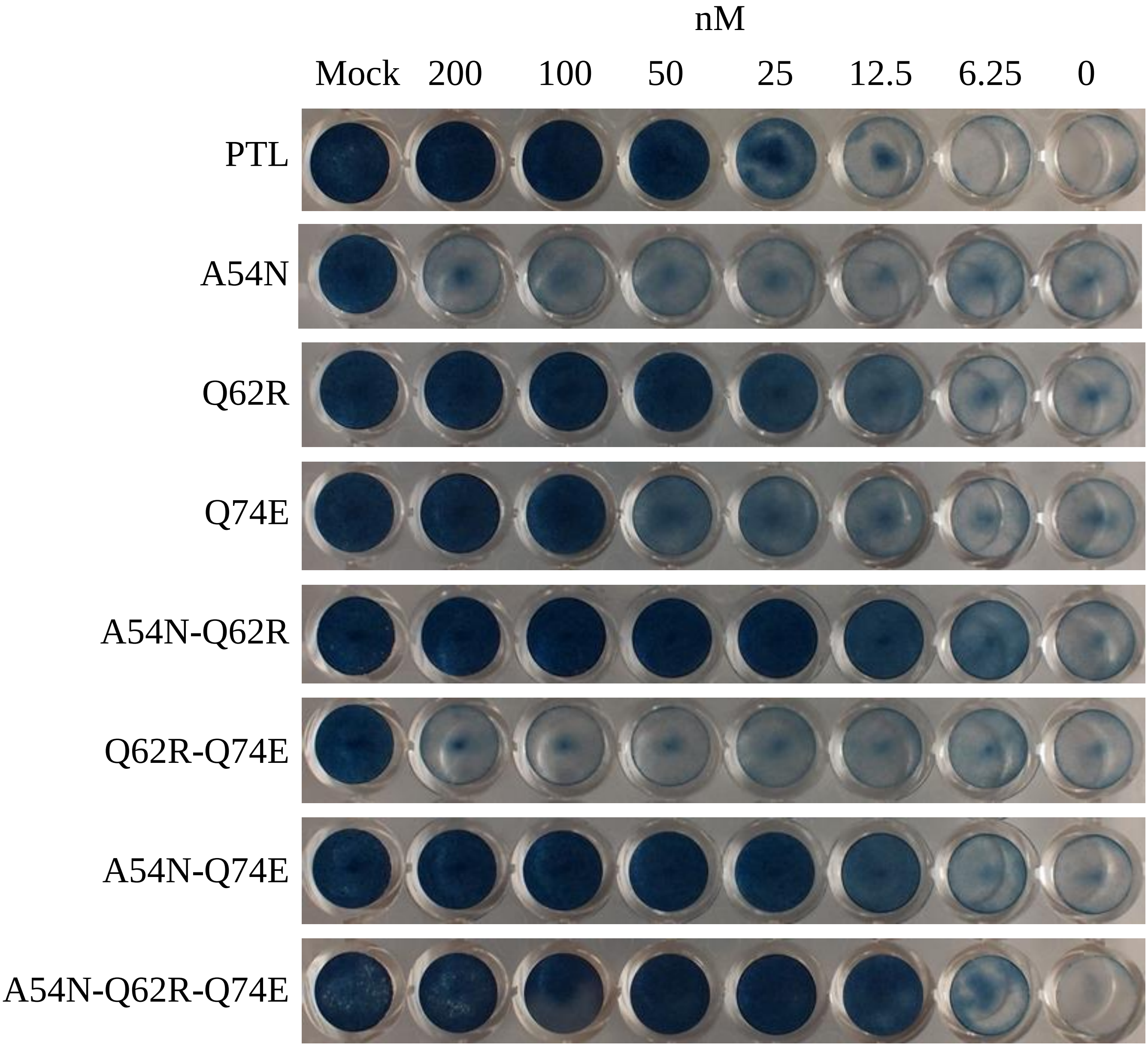

Fig. S1. Representative pictures of stained infected culture plates. Each data point in **Fig. 5** was obtained from averages of at least four measurements from different plates. Mock indicates no virus infection with 200 nM of lectin.
